# C-reactive protein elevation ratio as an early predictor of postoperative severe complications after laparoscopic gastrectomy for gastric cancer: a retrospective study

**DOI:** 10.1186/s12893-019-0582-9

**Published:** 2019-08-20

**Authors:** Hiroaki Tanaka, Tatsuro Tamura, Takahiro Toyokawa, Kazuya Muguruma, Naoshi Kubo, Katsunobu Sakurai, Masaichi Ohira

**Affiliations:** Department of Gastroenterological Surgery, Osaka City University Graduate School of Medicine, 1-4-3 Asahi-machi, Abeno-ku, Osaka, 545-8585 Japan

**Keywords:** Laparoscopic gastrectomy, Postoperative complication, C-reactive protein, Gastric cancer

## Abstract

**Background:**

In gastrectomy, postoperative elevation of C-reactive protein (CRP) is thought to be useful for predicting complications. Laparoscopic gastrectomy (LG) is less invasive than laparotomy and the elevation of CRP is also mild. Postoperative complications such as anastomotic leakage not only increase the severity of the condition, but also carry a poor prognosis when treatment is delayed. Early treatment is therefore necessary.

**Method:**

This retrospective study examined the relationship between occurrence of complications and the ratio of CRP levels on postoperative days 1 and 3 (CRP ratio) for 449 gastric cancer patients who underwent LG in the Department of Gastrointestinal Surgery at Osaka City University Hospital between 2006 and 2016.

**Results:**

We observed that factors associated with postoperative complications were preoperative renal failure and CRP ratio. No significant associations with surgical procedure, operation time, bleeding volume, age, obesity, measured CRP concentration, or white blood cell count were evident. The optimal cut-off for CRP ratio to predict postoperative complications from the receiver operating characteristic curve was 2.13.

**Conclusion:**

Our results suggested that the risk of severe postoperative complications after LG could be predicted using the CRP ratio.

## Introduction

Laparoscopic gastrectomy (LG) is widely accepted in East Asia as a minimally invasive surgery for gastric cancer [1]. The benefits of LG include reduced surgical trauma, pain and quick recovery [2, 3]. Postoperative complications, however, still occur even after LG, and the overall postoperative complication rate after gastrectomy has been reported as 7–28% [4–6]. Surgeons should recognize the risk of complications before the presentation of overt symptoms and seek early diagnosis, because complications after gastrectomy may adversely affect long- and short-term outcomes in patients [7–9]. With regard to gastrectomy, several investigators have reported a relationship between postoperative C-reactive protein elevation and complications [10–13]. However, those reports analyzed values from datasets that included open gastrectomy (OG). Suitable CRP-related values to predict postoperative complications after LG thus remain uncertain. We hypothesized that postoperatively elevated CRP concentrations would be predictive of postoperative complications after LG.

The aim of this retrospective study was to investigate the elevation rate of CRP as an early predictor of postoperative complications after LG for gastric cancer.

## Patients and methods

### Patients and data collection

Data were obtained from 449 patients with histologically confirmed gastric cancer who had undergone LG with lymph node dissection between 2006 and 2016 in the Department of Surgical Oncology at Osaka City University. Stage and other information including macroscopic type, histology, depth of tumor (T), lymph node metastasis (N), distant metastasis (M), lavage cytology (CY), and lymphatic or venous involvement were classified according to the criteria of the 3rd edition of Japanese Classification of Gastric Carcinoma by the Japanese Gastric Cancer Association. Data for clinicopathological characteristics, intra-operative findings, and postoperative course were extracted from our database. The Clavien-Dindo (CD) classification was adopted for evaluating postoperative complications [14]. Complications were defined as those of CD grade II or higher, with complications of grade IIIa or higher considered severe. This retrospective study was approved by the ethics committee of Osaka City University (#4038) and carried out according to the Declaration of Helsinki. We collected laboratory data including white blood cell count (WBC), neutrophil count, lymphocyte count, and CRP concentration on postoperative days (POD)1 and POD3. The CRP ratio was defined as: (CRP concentration on POD3) / (CRP concentration on POD1).

### Statistical analysis

Relationships between complications and other variables were analyzed using the Mann-Whitney U test. Uni- and multivariate logistic regression analyses were performed. Values of *P* < 0.05 were considered statistically significant. The cut-off value for the CRP ratio was determined using receiver operating characteristic (ROC) curves. Analysis was performed using JMP® version 11 software (SAS Institute, Cary, NC). The area under the curve and 95% confidence interval of the ROC curve and the power of this study were calculated using EZR (Saitama Medical Center, Jichi Medical University, Saitama, Japan), a graphical user interface for R (The R Foundation for Statistical Computing, Vienna, Austria). More precisely, EZR is a modified version of R Commander designed to add statistical functions frequently used in biostatistics [15].

## Results

### Patient characteristics

Background characteristics of the cohort in this study are summarized in Table 1. Of the 449 patients, 41 (9.1%) were more than 80 years old, and 57 (12.6%) had a body mass index > 25 kg/m^2^. Some form of comorbidity was identified in 77 patients (17.1%), including diabetes mellitus, renal dysfunction, ischemic heart disease, brain infarction, liver cirrhosis, and chronic obstructive pulmonary disease. Twenty patients showed multiple comorbidities. Distal gastrectomy was performed for 393 patients (87.5%), and D2 lymph node dissection for 48 patients (10.6%). Mean intraoperative blood loss was 121 g and mean operation time was 283 min. Pathological results were stage I in 389 (86.6%) patients, stage II in 41 (9.3%), and stage III in 19 (4.2%). Median white blood cell count was 10,000/mm^3^ on POD1 and 7000/mm^3^ on POD3, and median CRP concentration was 5.8 mg/dL on POD1 and 7.3 mg/dL on POD3.

**Table 1:** Patients characteristics and difference between severe complitcation and no or mild complication

	Total patients (*n* = 449)	Severe Complication (*n* = 33)	No or mild complication (*n* = 416)	*p* value
Age				
≤ 80	408	29	379	0.535
≤ 81	41	4	37	
Sex				
Male	299	27	272	0.325
Female	150	6	144	
BMI				
≤ 25	392	27	365	0.325
> 25	57	6	51	
Preoperative comorbidity				
DM	62	10	52	0.004
Renal dysfuction	5	3	2	< 0.001
Ischemic heart disease	28	4	24	0.146
Brain infarction	25	2	23	0.898
Liver cirrhosis	5	0	5	0.526
COPD	14	1	13	0.976
Multiple Comorbidities	20	3	17	0.011
Operation time				
Average (min)	281	280	283	0.813
Blood loss				
Average (g)	121	124	121	0.936
Surgery				
DG	393	26	367	0.145
TG	56	7	49	
Lymphatic dissection				
D1, D1+	401	32	369	0.334
D2	48	1	47	
pathological T category				
pT1,2	411	26	385	0.012
pT3,4	38	7	31	
pathological N category				
pN0	385	25	360	0.302
pN1,2,3	64	8	56	
pathological Stage				
Stage I	389	25	364	0.005
Stage II,III	60	8	52	
Laboratory data				
Day1 (Median)				
WBC (×10/mm3)	100	101	99	0.186
CRP (mg/dL)	5.75	4.5	6.3	0.057
Neutrophils (%)	83.8	84.3	83.6	0.243
Lymphocyte (%)	10.5	10	11	0.954
Day3 (Median)				
WBC (×10/mm3)	70	85	68	0.373
CRP (mg/dL)	7.3	13.5	6.3	0.671
Neutrophils (%)	79.4	78.7	49.1	0.501
Lymphocyte (%)	15	12.3	15.5	0.079
Difference (Day3-Day1)				
DWBC (×10/mm3)	−28	−19	−32	0.483
DCRP	1.39	9	0.4	0.113
Dneutrophils (×10/mm3)	−31	−20	−31	0.741
Ratio (Day3/Day1)				
(Median)				
WBC	0.7	0.8	0.7	0.844
CRP	1.28	2.9	1.1	0.003
Neutrophils	0.6	0.7	0.58	0.768

### Association of postoperative complications with perioperative background

Postoperative complications are summarized in Table 2. Minor complications (less than CD grade II) included wound site infection, elevated CRP, fever, and intestinal paralysis. Most instances of infectious complications, such as anastomotic leakage and pancreatic fistula, were CD grade III or more. Severe complications (CD grade III or more) occurred in 33 patients (7.3%) (17 cases of anastomotic leakage, 6 cases of pancreatic fistula, 4 cases of intraabdominal abscess, 2 cases of aspiration pneumonia, and 2 cases of strangulation ileus). Three patients showed multiple complications of anastomotic leakage and pancreatic fistula. One patient with CD Grade 5 complications died of cardiac infarction.

**Table 2:** Postoperative compications

	No complication	Clavien-Dindo classification						
		I	II	IIIa	IIIb	IVa	IVb	V
total	358	24	34	22	1	5	2	3
Anastomotic leakage	0	0	1	11	1	2	2	1
Pancreatic fistula	0	0	8	6	0	0	0	0
Intraabdominal abscess	0	0	1	4	0	0	0	0
Aspiration Pneumonia	0	0	0	1	0	1	0	1
Strangulation ileus	0	0	0	0	0	2	0	0
Bleeding	0	0	0	2	0	1	0	0
Other	0	24	24	1	0	0	0	1

We observed no associations of gender, age, or obesity with postoperative complications (Table 1). Complication rates were high in patients with diabetes, patients with renal dysfunction requiring artificial dialysis, and patients with multiple comorbidities, ranging from 15 to 52%. We found no statistical differences in complication rates between patients after laparoscopic total gastrectomy (LTG) and laparoscopic distal gastrectomy (LDG). Anastomotic leakage was observed in 8 cases of LTG and 10 cases of LDG. Although the amount of intraoperative blood loss, operation time, and lymph node dissection showed no correlations, complications occurred frequently in cases where the pathological TNM stage was II/III.

### Impact of postoperative laboratory data on complications and predictive value of CRP ratio

No factors in postoperative blood data showed associations with complications, including CRP, white blood cells, and neutrophils, which were considered as markers of bacterial infection. Since blood test data were obtained on POD1 and POD3, the difference and rate of change were calculated and correlations with complications were investigated. No association was identified between changes in WBC, CRP, or neutrophil count, but an association was identified between the CRP ratio and complications (Table 1). Multivariate analysis identified pathological stage, multiple comorbidities, and CRP ratio as independent predictors of severe postoperative complications (Table 3).

**Table 3:** Multivariate analysis

	Standard error	Wald-square	*p* value	Odds ratio	95% CI
CRP ratio	0.33	11.393	0.001	3.67	1.7300–7.860
pStage	0.386	8.195	0.004	5.6	1.710–18.400
Multiple Comobidities	0.852	10.63	0.004	3.27	1.470–7.280
R2 = 0.117 Pr > ChiSq 0.0002	Goodness-of-fit statistics Pr > ChiSq 0.343				

**Fig. 1:**
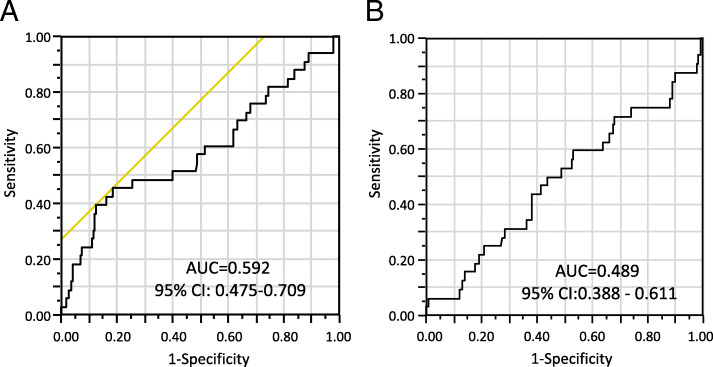
Receiver operating characteristic (ROC) curves for CRP ratio and CRP on postoperative day 3. We used the continuous variable density of CRP ratio (**a**) and CRP on postoperative day 3 (**b**) as the test variable and postoperative complications as the state variable. Area under ROC curve was 0.592 for CRP ratio and 0.498 for CRP on postoperative day 3. An investigation of cut-off scores showed the optimal cut-off for CRP ratio was 2.13 (sensitivity, 0.454; specificity, 0.815). We therefore set 2.13 as the cut-off for CRP ratio

To determine the optimal cut-off for CRP ratio, we analyzed the ROC curve (Fig. 1a). A cut-off CRP ratio of 2.13 offered 55% sensitivity and 82% specificity for postoperative severe complication, representing the optimal cut-off. The area under curve (AUC) was as low as 0.592, but in comparison, the AUC for CRP on POD3 was 0.489 (Fig. 1b). We then divided patients into two groups: high CRP ratio (≥2.1), 92 patients (20%); and low CRP ratio (< 2.1), 357 patients (80%) (Table 4). Of the 357 patients, 339 showed no complications after surgery, indicating a negative predictive value of 94%. On the other hand, the positive predictive value for postoperative complications was 16%.

**Table 4:** Predicitive valuse according to CRP elevation ratio

	CRP elevation ratio	
	≥2.13 (*n* = 92)	<2.13 (*n* = 357)
Severe Complication	15	18
No or mild comprication	77	339

## Discussion

This study showed a significant association between postoperative CRP elevation and severe postoperative complications after LG. Moreover, CRP ratio, defined here as the ratio of CRP levels on POD1 and POD3, represented an independent risk factor for severe postoperative complications.

LG offers various advantages over OG, including shorter duration of hospitalization, reduced blood loss, faster recovery of bowel movement, and earlier ambulation [16]. An updated meta-analysis found no significant difference in postoperative complication rates between LG and OG groups for advanced gastric cancer [17]. Postoperative complications reportedly occurred in 9.8–12.7% of LG patients with stage I or II cancer in two Asian studies [17, 18]. We observed that the complication rate was 14.9% for CD grade II or more and 7.3% for CD grade III or more in the present study. Postoperative intra-abdominal infectious complications such as anastomotic leakage, pancreatic fistula, and intraperitoneal abscess increase surgical stress and cause severe tissue damage due to local and generalized inflammatory reactions, resulting in more severe immune suppression [9].

Numerous studies have identified postoperative CRP concentration as a risk factor for postoperative complications after gastrointestinal cancer surgery, including colorectal cancer, esophageal cancer, and gastric cancer [12, 19–21]. In minimally invasive surgery and laparotomy for colorectal and gastric cancer, postoperative CRP levels have been observed to be significantly lower after minimally invasive surgery if no complications arise [22–24]. Many previous reports on the association between CRP and postoperative infectious complications have involved a mixture of laparotomy and laparoscopic surgery. Of the few reports that have focused on laparoscopic surgery, 300 to 400 cases were examined, with LG accounting for less than 50% of those cases [10, 12, 19]. No marked difference was seen between CRP levels in those reports and levels in laparotomy cases only, and peak CRP concentration is usually observed on POD3 or POD4, with a threshold for predicting complications of 12–17.7 mg/dL [11–13, 25]. Although fewer than 300 cases have previously been reported when limited to laparoscopic surgery, 440 cases were reported in the present study. The significance of this report is considered to be equivalent to that of other reports.

Elevated CRP as a predictor of postoperative complications has usually been examined at a single time point. In this study, however, CRP levels on POD3 alone were not associated with complications. One reason for this was likely to be the fact that we examined patients with serious complications other than infectious complications, such as cases requiring reoperation due to severe adhesive ileus, cases of shock due to postoperative bleeding, and cases of death due to myocardial infarction early after surgery. Moreover, high CRP levels were associated with long operation time, greater blood loss, and high BMI. We observed a CRP concentration > 17.5 mg/dL on POD3 in 10% of patients without complications. We therefore focused on the “CRP ratio” to predict postoperative complications. Another reason for using the CRP ratio in this study was the difficulty of setting cut-off values. CRP levels on POD3 were elevated to the same level in both LG and OG groups with and without complications after colorectal cancer surgery, and CRP levels on POD3 were not final predictors of complications [23]. Elevated CRP levels ≥10 mg/dL are generally considered clinically indicative of marked inflammation, but the wide range of cut-off values reported previously can make clinical application of such findings difficult [26]. We showed a negative predictive value of 94% for CRP ratio in this study. As the number of minimally invasive surgeries continues to increase, the length of hospital stays may be shortened. A recently published meta-analysis reported the importance of CRP level on POD3 in predicting readmission after radical gastrectomy [27]. Those results suggest that the CRP elevation rate after surgery may be useful for safely determining early discharge.

Several limitations to this study must be considered. This was a single-center, retrospective, observational study and the sample size was small. The power of this study was relatively low, at 0.78, because of the small number of samples. This represents a substantial weakness in the statistical analysis. Validation studies using larger cohorts from multiple centers are needed to confirm the impact of CRP ratio on postoperative complications. Second, only short-term surgical outcomes were evaluated. Pancreatic fistula or intraperitoneal abscess appearing 2 weeks or more after surgery appear unlikely to be anticipated using data from POD3. Third, other parameters that were not assessed included the CRP-to-albumin ratio, estimated glomerular filtration rate, platelet-to-lymphocyte ratio, sarcopenia, and preoperative prealbumin concentration [28–32]. We observed that the complication rate was significantly higher in patients with renal failure on dialysis. Immune response, nutritional condition, and performance status should also be investigated. Despite these limitations, CRP ratio appears potentially useful as an easily understood value for clinical use.

## Conclusion

In conclusion, our findings suggested that CRP ratio after laparoscopic gastrectomy could offer a useful predictor of postoperative complications likely to need treatment. Our results suggest that unnecessary treatments such as drain replacement and antibiotics might be obviated when the CRP ratio is low on POD3 after LG.

## Data Availability

The datasets used and/or analysed during the current study are available from the corresponding author on reasonable request.

## Abbreviations

AUC: Area under curve
CD: Clavien-Dindo
CRP: C-reactive protein
LDG: Laparoscopic distal gastrectomy
LG: Laparoscopic gastrectomy
LTG: Laparoscopic total gastrectomy
OG: Open gastrectomy
POD: Postoperative day
ROC: Receiver operating characteristic
